# Repeatability of Intraoral Scanners for Complete Arch Scan of Partially Edentulous Dentitions: An In Vitro Study

**DOI:** 10.3390/jcm8081187

**Published:** 2019-08-08

**Authors:** Jae-Hyun Lee, Je-Hyeon Yun, Jung-Suk Han, In-Sung Luke Yeo, Hyung-In Yoon

**Affiliations:** 1 Department of Prosthodontics, One-Stop Specialty Center, Seoul National University Dental Hospital, Daehak-ro 101, Jongro-gu, Seoul 03080, Korea; 2 Department of Prosthodontics, School of Dentistry and Dental Research Institute, Seoul National University, Daehak-ro 101, Jongro-gu, Seoul 03080, Korea

**Keywords:** accuracy, digital dentistry, intraoral scanner, partial edentulism, precision

## Abstract

Research on whether the number or location of missing teeth affects the accuracy of intraoral scanners in partial edentulous patients is scarce. This study aimed to evaluate the precision of complete-arch scan data of various partial edentulous arches acquired by intraoral scanners. Five different maxillary models were scanned using Carestream CS3600 and Medit i500 scanners. The models employed here were control: Fully dentate; Case 1: Missing a right second premolar and a first molar; Case 2: Missing a right second premolar, a first molar, both left premolars, and a left first molar; Case 3: Missing four incisors and a right canine; and Case 4: Missing four incisors, a left second premolar, and a first molar. Six scans per group were performed and the resulting two datasets were paired to analyze the precision of each group (*n* = 15). Two-way ANOVA was performed (α = 0.05). The root mean square (RMS) error values in Cases 2, 3, and 4 were significantly higher than those in Case 1 and control. The RMS values of the two intraoral scanners were not significantly different. Scanning precision was significantly lower for both devices when used for scanning dental arches with ≥5 missing teeth.

## 1. Introduction

The use of digital methods, such as computer-aided design and computer-aided manufacturing (CAD–CAM), is rapidly increasing in the field of dentistry [1,2,3,4]. Research related to and applications of digital dentistry are being actively conducted and explored in almost all dental fields, such as restorative and surgical dentistry and orthodontics [3,4,5]. Recently, with the development of CAD–CAM equipment and various new materials, chairside fabrication methods are being more widely used [6,7,8]. However, to effectively perform many dental procedures using a completely digital workflow without producing conventional stone casts, any intraoral scanners employed should clearly achieve clinically acceptable levels of accuracy [9,10].

The accuracy of such devices is largely evaluated in two ways: Trueness and precision [11,12,13]. Trueness is an estimate of how close a measured value is to “the truth” and precision is a measure of how closely the measured values match each other [12]. Precision is again referred to in two ways: Repeatability, which usually refers to the precision involved when the same operator measures the same object multiple times in the same environment, and reproducibility, which is evaluated by the degree to which the results are consistent even when different operators are doing the scanning or when the scanning environment changes [11].

There have been several research efforts to evaluate the accuracy of a wide range of intraoral scanners [9,14,15,16,17,18,19,20,21,22,23]. Comparisons regarding the accuracy of such devices from different manufacturers were previously reported [11,20]. Furthermore, the differences in accuracy between scanning partial arches and full arches were investigated [14]. Studies were also conducted on the accuracy of intraoral scans for completely edentulous arches [24,25]. However, there have been few rigorous studies addressing the accuracy of scanning partially edentulous arches [26,27].

There are many cases in which a partially edentulous patient is treated during routine dental practices. In such situations, a tooth loss site can be rehabilitated not only with a removable partial denture, but also using a fixed partial denture, which can be fine-tuned through orthodontic treatment. Moreover, when restoring the edentulous area with dental implants, at the diagnosis stage before the implant fixture is installed, an intraoral scan of the edentulous part is required without the help of scan bodies. To conduct a modeless dental practice to rehabilitate partially edentulous cases, verification of the accuracy of intraoral scanners is essential. In particular, partially edentulous arches may present a wide array of scenarios depending on the number and location of the missing teeth, which makes it necessary to determine whether there is a difference in accuracy according to the various cases of partially edentulous dentitions.

In view of this scientific lacuna, the present study aimed to evaluate the precision of complete-arch scan data of various partially edentulous states acquired by two intraoral scanners. The first null hypothesis of this study was that various cases of partially edentulous dentitions would not affect the complete-arch scan data precision of the intraoral scanners. The second null hypothesis was that, when scanning partially edentulous dentitions, no significant difference would be found in repeatability between the two devices from two different manufacturers.

## 2. Materials and Methods

### 2.1. Evaluated Scenarios

**Figure 1:**
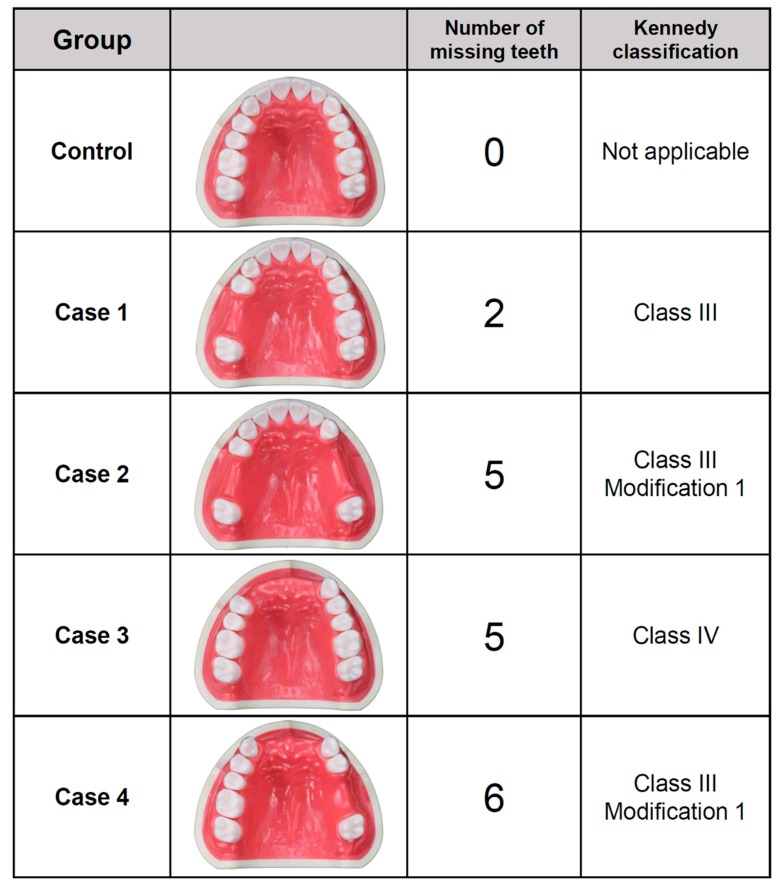
Classification of maxillary models for the scan. The simulated soft tissue area was fabricated from hard acrylic resin material.

Five commercially available maxillary dentiform models (500A, 523, 573, 583, and 543; Nissin, Tokyo, Japan), which represented five oral conditions, were used in the present study (Figure 1). One was a maxillary, fully dentate model with no edentulous space and was used as a “control”. The other four represented partially edentulous dentitions of the Kennedy classification III or IV. Case 1 was a Kennedy classification III, with two missing posterior teeth: A maxillary right second premolar and a first molar. Case 2 was a Kennedy classification III modification 1, with five missing posterior teeth: A maxillary right second premolar, a first molar, both left premolars, and a left first molar. Case 3 was a Kennedy classification IV, with five missing anterior teeth: Four maxillary incisors and a right canine. Case 4 was a Kennedy classification III modification 1, with six missing anterior and posterior teeth: Four maxillary incisors, a left second premolar, and a first molar. The simulated edentulous ridge areas of all the dentiform models were manufactured using gingival-colored acrylic resin, which was the same material used to fabricate the artificial teeth of the dentiform models and differed only in color. Silicone rubber materials were not used for the simulated soft tissue areas.

### 2.2. Use of Intraoral Scanners

Two types of intraoral scanners, the CS3600 (version 3.1.0; Carestream Dental, Atlanta, GA, USA) and the i500 (iScan version 1.2.0.1; Medit, Seoul, Korea), were used to obtain complete-arch digital impressions of the five models. A single operator (JHY), who was proficient in using both scanners, performed all the image acquisition procedures. Six repeated complete-arch scans of each model were conducted using each of the scanners. By selecting two of these six scans, 15 pairs from each group could be analyzed (*n* = 15). The number of repeated scans was determined by a power analysis (actual power = 98.3%; effect size *f* = 0.4; α = 0.05; power = 0.95) using G*Power (version 3.1.9.4 Universität Kiel, Kiel, Germany) [28]. When imaging the fully dentate version, the scan was started from the second molar on the right side. The partially edentulous models were scanned, beginning at the second molar with the smaller edentulous side between the second molars on both sides. No powder was used during the scanning procedures. The scanning procedures were followed according to the guidelines of the manufacturers. For each scanning, the acquisition type of the CS3600 was set as a restoration mode. Each scanning procedure with i500 was conducted under the recommended conditions with level-2 filtering and a 17.0-mm scan depth. The scanning time was approximately 120 s for each arch and the procedures were performed such that no data were missed. After all the digital scans were acquired, they were exported as standard tessellation language (STL) files and the water-tightness (meaning that the datasets had no missing observations [11]) of the digital models was confirmed.

### 2.3. Precision Assessment

Precision was determined in accordance with how the International Organization for Standardization (ISO, Geneva, Switzerland) defines this concept (ISO 5725-1) [12]. To evaluate the precision of each group, pairwise comparisons among the six scans of each group were performed and, by selecting two of these six scans, a total of 15 pairs from each group were analyzed (*n* = 15). Uninterested areas of the imported images were removed before measurements. The 3-dimensional (3D) superimpositions of the paired STL datasets were conducted using the best-fit algorithm of an inspection software system (Geomagic Control X, version 2017.0.3; 3D Systems, Cary, NC, USA). The alignment algorithm matches the coordinates of the target data to the deviation between geometric shapes that is minimized within an allowable tolerance. In this study, point-to-point matching and the iterative closest point algorithm were used for each inspection procedure. After an initial alignment, the least-squares method (to minimize the sums of squares of distances between the cloud of points) was used for best-fit alignment between the scan data. Each inspection was performed under a sampling ratio of 100% and a maximum iteration count of 100. All inspection procedures were conducted under the guidance of a 3D Systems engineer. The resulting root mean square (RMS) error values were then documented to represent the amount of deviation [26,27,29]. The RMS error value between scan data was calculated by applying following formula:

(1) $$\mathrm{RMS}=\;\frac{\sqrt{\sum_{i=1}^{n}{\left(X_{1,i}-X_{2,i}\right)}^{2}}}{\sqrt{n}}$$

where, *n* is the total number of points of measurement, $X_{1,i}$ is the value of measurement of point i on the first scan data, and *X*_2,*i*_ is the value of measurement of point *i* on the second scan data. Based on the superimposition analysis, the color maps of the 3-dimensional surface deviation were obtained, with a nominal value of 0 μm and a critical value of ±200 μm.

### 2.4. Statistical Analysis

All statistical analyses were performed using SPSS, version 24 (IBM Corp., New York, NY, USA). Two-way ANOVA tests were conducted to determine the effect of two factors on the RMS values: The various cases we created and the different intraoral scanners. Analyses of the interactions and a Bonferroni correction were performed. The statistical significance was set at 0.05. To determine the power of the sample size, a post hoc power analysis was also conducted.

## 3. Results

Table 1 shows the outcome of our two-way ANOVA tests. It reveals that the RMS values varied considerably across the five different dentitions (*p* < 0.001, post hoc power = 1.00). However, the type of intraoral scanners did not have a significant impact on precision (*p* = 0.057). Also, no significant interaction was found between the intraoral scanners used and the dentitions evaluated (*p* = 0.901).

**Figure 2:**
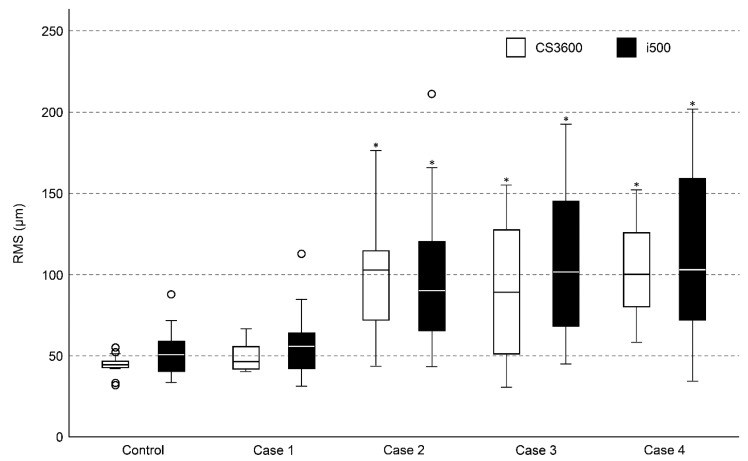
Box plot demonstrating precision measurement. The amount of deviation is represented as root mean square (RMS). * Significantly different from control group scanned with the same intraoral scanner (*p* ≤ 0.05).

Table 2 and Figure 2 present the mean values and distribution of each group. For both of the intraoral scanners, the complete-arch scan data of the fully dentate arch with no edentulous space (control) showed the least RMS value. The RMS value of Case 1 (short-span edentulous space) was not significantly different from that of the control group. By contrast, Cases 2, 3, and 4 (long-span or multiple edentulous spaces) presented significantly higher RMS values than the control, regardless of the scanner. No significant differences were observed among the RMS values of Case 2, 3, and 4.

**Figure 3:**
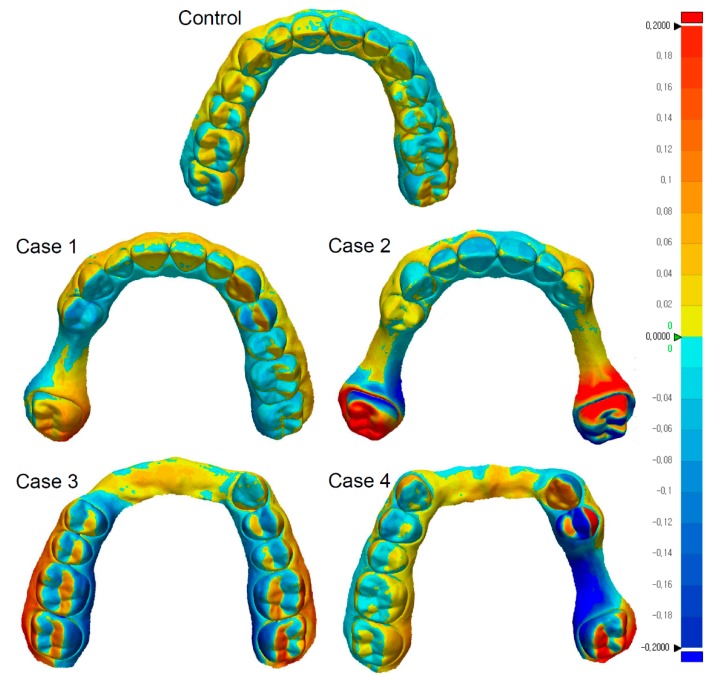
Representative images from a color map for assessing precision. The color maps for the CS3600 datasets are displayed to visualize the distribution of deviation. Deviation sites are mainly observed on the side that includes the partially edentulous area.

When comparing the precision of the intraoral scanners, a statistically significant difference was not observed between the two intraoral scanners for any of these cases. Both of the scanners produced similar trends according to the evaluated dentition status. The representative images of color maps are displayed in Figure 3, clearly showing the 3D surface deviation in the complete-arch scan data of the partially-edentulous dentitions. The distribution pattern of 3D surface deviation in the color map of Case 1 was visually similar to that of the control group, but it differed from that of other groups. Deviation sites were mainly observed around the partially edentulous areas.

## 4. Discussion

This in vitro study investigated the influences of the location and number of missing teeth on the precision of complete-arch by two different intraoral scanners. Our first null hypothesis was rejected because the type of dentition clearly affects the repeatability of the intraoral scanners at a power greater than 95%. In contrast, our second hypothesis was accepted because there was no significant difference in the performance of the two intraoral scanners when scanning partially edentulous dentitions. Case 1, which had a relatively small number of missing teeth, had RMS values similar to those of the control group. However, Cases 2, 3, and 4, all of which had a large number of missing teeth, had significantly larger RMS values than the control group did. No significant differences were observed between the two intraoral scanners in any dentition case in terms of RMS values. Therefore, the precision of the scan results can vary according to the type of partial edentulous dentitions, irrespective of the type of intraoral scanner being used.

To the best of our knowledge, no previous study investigated the effects of the varying extent of Kennedy classifications III or IV edentulous areas on the accuracy of intraoral scanning. Previously, Hayama et al. [26] studied the association between partial edentulism, using Kennedy classifications I and III, and intraoral scanning accuracy. However, they mainly analyzed the accuracy of the various scanner head sizes and impression methods regarding partial edentulous dentitions and did not specifically analyze the effects of different edentulous forms [26]. In addition, that team scanned models with simulated mucosa made of deformable silicone material [26]. In our investigation, the mucosal areas of our models were fabricated with the same hard resin material used to make artificial teeth. The reason for choosing the specific models in this study was to rigorously exclude the difference in impression results due to the characteristics of the elastic material employed, such as silicone. Because we could rule out the inaccuracy of scanning due to deformation of the simulated mucosal area, it can be stated that the repeatability of the scan was different according to the geometrical shape of the dentition, except the fluctuation due to the flexibility of the soft-tissue area. Therefore, it was concluded that the precision of the complete-arch scan data varied with the length and distribution of the edentulous area.

The primary reason for the increase in errors when addressing partially edentulous dentitions could be due to the image acquisition method of the intraoral scanners. These devices acquire scan data by stitching together images using a complex best-fit algorithm. To properly align the many images involved, it turns out to be advantageous for the scanned object to have a complex geometric shape. If the digitized item is too simple, an error may occur in the process of aligning the images [11]. Therefore, when scanning a relatively flat and smooth toothless region, errors in the alignment of scan data may be greater than when scanning an area having a much more complex shape, such as the occlusal surface of a tooth [11]. Perhaps, owing to these reasons, the deviations were more concentrated on the side that included the partially edentulous area than on the side without missing teeth, as displayed in Figure 3.

Among the various partially edentulous dentitions evaluated in this study, Case 1, in which only two teeth were lost, produced no significant difference in precision compared to the control group. However, Cases 2, 3, and 4, with more than five teeth missing, significantly decreased the repeatability of the oral scanners compared to the control group, clearly showing that the accuracy of the intraoral scan decreased as the edentulous area increased in partially edentulous dentitions. This reality may be important not only when restoring a partially edentulous area with a removable partial denture, but also when fabricating a fixed partial denture to restore a long-span edentulous area or to make a computer-guided stent to prepare for implant surgery. The largest mean RMS value in this study was 115.7 ± 53.3 µm. This RMS value is similar to the previously reported clinically acceptable margin gap value (120 µm) for fixed dental restorations [30]. To minimize the potential ill-fitting problem, it may be helpful to temporarily attach geometrically complex objects in the middle of the long edentulous site. Indeed, this straightforward method was previously reported to improve accuracy in the scanning of edentulous areas [29].

The limitation of this study was that the accuracy of the complete-arch scan data was only evaluated for repeatability or precision of the intraoral scan. This study was designed to obtain experimental values for use in comparative analysis with measurements that will be obtained from future in vivo studies evaluating the precision of complete-arch data obtained from intraoral scanning of partial edentulous dentitions. In addition, the in vitro evaluation performed in this work may not fully simulate the clinical practice environment or condition. The precision of an intraoral scan can be affected by many aspects of the intraoral environment, such as saliva, moisture, temperature, and movement of the soft-tissue area. Therefore, it is necessary to assess how the accuracy of intraoral scanners varies based on the position, number, and size of the toothless area in the dental arch. Additionally, the results of the present study require confirmation via future clinical studies.

## 5. Conclusions

Within the limitation of this in vitro study using models employing a hard mucosal area, the following conclusions can be drawn:

1. In the scanning of partially edentulous dentitions, the repeatability of complete-arch scan data acquired by intraoral scanners was reduced in the cases having relatively large edentulous sites;
2. Surface deviations were mainly observed on the side that included the partially edentulous area in the scanned dental arch;
3. Regardless of the type of intraoral scanners used, a similar trend was observed in the repeatability of complete-arch scan data obtained for partially edentulous dentitions.

Dentists should be aware that, even with careful attention to movement or deformation of the soft tissue area when scanning partially edentulous patients with large or multiple missing sites, the precision of virtual models may be reduced due to the functional limitations of the currently available intraoral scanners.

## Figures and Tables

**Table 1:** Results of two-way ANOVA with the root mean square (RMS) as the dependent variable.

Source	Type III Sum of Squares	*df*	Mean Squares	*F*	*p*
Dependent variable: RMS					
Scanner	4408.63	1	4408.63	3.68	0.057
Case	94,020.02	4	23,505.00	19.61 ***	<0.001
Scanner × Case	1262.79	4	315.70	0.26	0.901
Error	167,795.36	140	1198.54		

*** *p* < 0.001.

**Table 2:** Mean and standard deviation (SD) of root mean square (RMS) values.

Group	RMS (unit: µm)				
	CS3600		i500		*p* *
	Mean	SD	Mean	SD	
**Control**	44.37 ^a^	6.04	52.30 ^a^	14.99	0.53
Case 1	49.57 ^a^	9.00	58.43 ^a^	21.45	0.48
Case 2	96.31 ^b^	35.29	100.22 ^b^	44.74	0.76
Case 3	85.59 ^b^	41.85	106.71 ^b^	48.92	0.10
Case 4	103.28 ^b^	30.86	115.66 ^b^	53.28	0.33

Bonferroni: a < b. Means with the same superscript in each column are not significantly different from each other based on the Bonferroni test (*p* > 0.05). * *p*-value between CS3600 and i500.

## References

[1] Blatz M.B., Conejo J. (2019). The Current State of Chairside Digital Dentistry and Materials. Dent. Clin. N. Am..

[2] Cervino G., Fiorillo L., Arzukanyan A.V., Spagnuolo G., Cicciu M. (2019). Dental Restorative Digital Workflow: Digital Smile Design from Aesthetic to Function. Dent. J. (Basel).

[3] Blackwell E., Nesbit M., Petridis H. (2017). Survey on the use of CAD-CAM technology by UK and Irish dental technicians. Br. Dent. J..

[4] Tran D., Nesbit M., Petridis H. (2016). Survey of UK dentists regarding the use of CAD/CAM technology. Br. Dent. J..

[5] Herford A.S., Miller M., Lauritano F., Cervino G., Signorino F., Maiorana C. (2017). The use of virtual surgical planning and navigation in the treatment of orbital trauma. Chin. J. Traumatol..

[6] Kaizer M.R., Gierthmuehlen P.C., Dos Santos M.B., Cava S.S., Zhang Y. (2017). Speed sintering translucent zirconia for chairside one-visit dental restorations: Optical, mechanical, and wear characteristics. Ceram. Int..

[7] Lambert H., Durand J.C., Jacquot B., Fages M. (2017). Dental biomaterials for chairside CAD/CAM: State of the art. J. Adv. Prosthodont..

[8] Nejatidanesh F., Savabi G., Amjadi M., Abbasi M., Savabi O. (2018). Five year clinical outcomes and survival of chairside CAD/CAM ceramic laminate veneers a retrospective study. J. Prosthodont. Res..

[9] Rutkunas V., Geciauskaite A., Jegelevicius D., Vaitiekunas M. (2017). Accuracy of digital implant impressions with intraoral scanners: A systematic review. Eur. J. Oral Implantol..

[10] Takeuchi Y., Koizumi H., Furuchi M., Sato Y., Ohkubo C., Matsumura H. (2018). Use of digital impression systems with intraoral scanners for fabricating restorations and fixed dental prostheses. J. Oral Sci..

[11] Braian M., Wennerberg A. (2019). Trueness and precision of 5 intraoral scanners for scanning edentulous and dentate complete-arch mandibular casts: A comparative in vitro study. J. Prosthet. Dent..

[12] International Organizationfor Standardization (1994). Accuracy (Trueness and Precision) of Measurement Methods and Results Part. 1: General Principles and Definitions.

[13] Latham J., Ludlow M., Mennito A., Kelly A., Evans Z., Renne W. (2019). Effect of scan pattern on complete-arch scans with 4 digital scanners. J. Prosthet. Dent..

[14] Ender A., Zimmermann M., Mehl A. (2019). Accuracy of complete and partial-arch impressions of actual intraoral scanning systems in vitro. Int. J. Comput. Dent..

[15] Fukazawa S., Odaira C., Kondo H. (2017). Investigation of accuracy and reproducibility of abutment position by intraoral scanners. J. Prosthodont. Res..

[16] Gan N., Xiong Y., Jiao T. (2016). Accuracy of Intraoral Digital Impressions for Whole Upper Jaws, Including Full Dentitions and Palatal Soft Tissues. PLoS ONE.

[17] Haddadi Y., Bahrami G., Isidor F. (2019). Accuracy of crowns based on digital intraoral scanning compared to conventional impression-a split-mouth randomised clinical study. Clin. Oral Investig..

[18] Kattadiyil M.T., Mursic Z., AlRumaih H., Goodacre C.J. (2014). Intraoral scanning of hard and soft tissues for partial removable dental prosthesis fabrication. J. Prosthet. Dent..

[19] Keul C., Guth J.F. (2019). Accuracy of full-arch digital impressions: An in vitro and in vivo comparison. Clin. Oral Investig..

[20] Kim R.J., Park J.M., Shim J.S. (2018). Accuracy of 9 intraoral scanners for complete-arch image acquisition: A qualitative and quantitative evaluation. J. Prosthet. Dent..

[21] Lim J.H., Park J.M., Kim M., Heo S.J., Myung J.Y. (2018). Comparison of digital intraoral scanner reproducibility and image trueness considering repetitive experience. J. Prosthet. Dent..

[22] Osnes C.A., Wu J.H., Venezia P., Ferrari M., Keeling A.J. (2019). Full arch precision of six intraoral scanners in vitro. J. Prosthodont. Res..

[23] Treesh J.C., Liacouras P.C., Taft R.M., Brooks D.I., Raiciulescu S., Ellert D.O., Grant G.T., Ye L. (2018). Complete-arch accuracy of intraoral scanners. J. Prosthet. Dent..

[24] Lo Russo L., Caradonna G., Troiano G., Salamini A., Guida L., Ciavarella D. (2019). Three-dimensional differences between intraoral scans and conventional impressions of edentulous jaws: A clinical study. J. Prosthet. Dent..

[25] Papaspyridakos P., Gallucci G.O., Chen C.J., Hanssen S., Naert I., Vandenberghe B. (2016). Digital versus conventional implant impressions for edentulous patients: Accuracy outcomes. Clin. Oral Implant. Res..

[26] Hayama H., Fueki K., Wadachi J., Wakabayashi N. (2018). Trueness and precision of digital impressions obtained using an intraoral scanner with different head size in the partially edentulous mandible. J. Prosthodont. Res..

[27] Marghalani A., Weber H.P., Finkelman M., Kudara Y., El Rafie K., Papaspyridakos P. (2018). Digital versus conventional implant impressions for partially edentulous arches: An evaluation of accuracy. J. Prosthet. Dent..

[28] Faul F., Erdfelder E., Lang A.G., Buchner A. (2007). G*Power 3: A flexible statistical power analysis program for the social, behavioral, and biomedical sciences. Behav. Res. Methods.

[29] Kim J.E., Amelya A., Shin Y., Shim J.S. (2017). Accuracy of intraoral digital impressions using an artificial landmark. J. Prosthet. Dent..

[30] Nawafleh N.A., Mack F., Evans J., Mackay J., Hatamleh M.M. (2013). Accuracy and reliability of methods to measure marginal adaptation of crowns and FDPs: A literature review. J. Prosthodont..

